# The crystal structures, Hirshfeld surface analyses and energy frameworks of two hexa­thia­pyrazino­phane regioisomers; 2,5,8,11,14,17-hexa­thia-[9.9](2,6,3,5)-pyrazino­phane and 2,5,8,11,14,17-hexa­thia-[9.9](2,5,3,6)-pyrazino­phane

**DOI:** 10.1107/S2056989020007057

**Published:** 2020-06-02

**Authors:** Tokouré Assoumatine, Helen Stoeckli-Evans

**Affiliations:** a Institute of Chemistry, University of Neuchâtel, Av. de Bellevax 51, CH-2000 Neuchâtel, Switzerland; b Institute of Physics, University of Neuchâtel, rue Emile-Argand 11, CH-2000 Neuchâtel, Switzerland

**Keywords:** crystal structure, regioisomers, *m*-bis­, *p*-bis­, thia­pyrazino­phanes, hexa­thia­pyrazino­phanes, Hirshfeld surface analysis, fingerprint plots, energy frameworks

## Abstract

The title thia­pyrazino­phanes, 2,5,8,11,14,17-hexa­thia-[9.9](2,6,3,5)-pyrazino­phane, C_16_H_24_N_2_S_6_, (**I**), and 2,5,8,11,14,17-hexa­thia-[9.9](2,5,3,6)-pyrazino­phane, C_16_H_24_N_2_S_6_, (**II**), are regioisomers; *m*-bis L1 and *p*-bis L1, respectively. Both compounds have a central tetra-2,3,5,6-methyl­ene­pyrazine unit with two –S—CH_2_—CH_2_—S—CH_2_—CH_2_—S– chains, linking the methyl­ene C atoms at positions 2 and 6 and 3 and 5 on the pyrazine ring of **I**, but linking the methyl­ene C atoms at positions 2 and 5 and 3 and 6 on the pyrazine ring of **II**. Both compounds crystallize with half a mol­ecule in the asymmetric unit. The whole mol­ecule of **I** is generated by inversion symmetry, with the pyrazine ring being located about a center of symmetry. The whole mol­ecule of **II** is generated by twofold rotation symmetry, with the pyrazine N atoms being located on the twofold rotation axis. In compound **I**, there are pairs of intra­molecular C—H⋯S contacts present, but none in compound **II**. In the crystal of **I**, there are no significant inter­molecular inter­actions present, while in the crystal of **II**, mol­ecules are linked by pairs of C—H⋯S hydrogen bonds, forming corrugated layers lying parallel the *ac* plane. The Hirshfeld surfaces and the energy frameworks of the two regioisomers indicate little difference in the inter­atomic contacts, which are dominated by dispersion forces.

## Abstract 2

The title hexa­thia­pyrazino­phanes are regioisomers, having a central tetra-2,3,5,6-methyl­ene­pyrazine unit with two –S—CH_2_—CH_2_—S—CH_2_—CH_2_—S– chains linking the methyl­ene C atoms at positions 2 and 6 and 3 and 5 in the *m*-bis regioisomer, but linking the methyl­ene C atoms at positions 2 and 5 and 3 and 6 in the *p*-bis regioisomer.

## Chemical context   

Ligands with mixed hard and soft binding characters, such as O, N and S donor atoms, are known to display diverse coordination modes by binding selectively to metal centres giving rise to unusual coordination geometries (Kim *et al.*, 2018 ▸; Klinga *et al.*, 1994 ▸; Lockhart *et al.*, 1992 ▸). Three regioisomers, *o*, *m* and *p*, of a bis-dioxadi­thia-benzeno­phane (L, O_4_S_4_) have been reported on by the group of Shim Sung Lee (Kim *et al.*, 2018 ▸). The structures of a number of metal complexes have also been described; for example, both *o*-bis L and *m*-bis L form one-dimensional coordination polymers with AgPF_6_ (Siewe *et al.*, 2014 ▸), while with lead(II) perchlorate a binuclear complex was obtained with *o*-bis L and a one-dimensional coordination polymer with *m*-bis L (Kim *et al.*, 2018 ▸). In all four complexes the metal atoms coordinate to both the O and S atoms.

The title compounds, **I** and **II**, are new N_*x*_S_*y*_ (*x* = 2, *y* = 2, 4 or 6) thia­pyrazino­phane ligands designed for the formation of coordination polymers (Assoumatine, 1999 ▸). We have recently reported on the crystal structures of two thia­pyrazino­phanes; the N_2_S_4_ ligand 3,4,8,10,11,13-hexa­hydro-1*H*,6*H*-bis­([1,4]di­thio­cino)[6,7-*b*:6′,7′-*e*]pyrazine (**L2**) and the N_2_S_2_ ligand 5,7-di­hydro-1*H*,3*H*-dithieno[3,4-*b*:3′,4′-*e*]pyrazine (**L3**) (Assoumatine & Stoeckli-Evans, 2020*a* ▸). On reaction of both **L2** and **L3** with AgNO_3_, two-dimensional coordination polymers were formed, with the silver(I) atoms coordinating to the S atoms only (Assoumatine & Stoeckli-Evans, 2020*a* ▸). On reaction of **L2** with CuI, a two-dimensional coordination polymer was formed with the ligand coordinating *via* the S atoms only (Assoumatine & Stoeckli-Evans, 2020*b* ▸). On reaction of **L3** with CuI, a three-dimensional coordination polymer was formed with the ligand coordinating *via* both the N and S atoms (Assoumatine & Stoeckli-Evans, 2020*c* ▸). Ligand **L3** was also shown to form one-dimensional coordination polymers with CuCl_2_ and CuBr_2_ (Assoumatine & Stoeckli-Evans, 2020*d* ▸), with the ligand coordinating *via* the N atoms only.
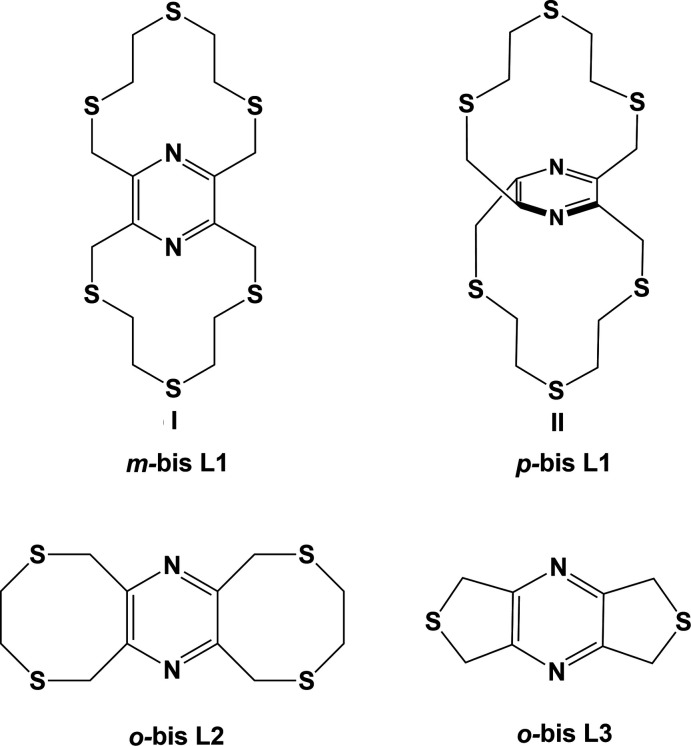


The coordination chemistry of the title compound *m*-bis L1 (**I**), an N_2_S_6_ thia­pyrazino­phane, has also been studied and shown to form a binuclear complex with CuBr_2_ and a two-dimensional coordination polymer with CuI (Assoumatine & Stoeckli-Evans, 2020*e* ▸). In both cases, the ligand coordinates to both the N and S atoms. Herein, we report on and compare the crystal structures, the Hirshfeld surfaces and the energy frameworks of the regioisomers *m*-bis L1 (**I**) and *p*-bis L1 (**II**).

## Structural commentary   

**Figure 1:**
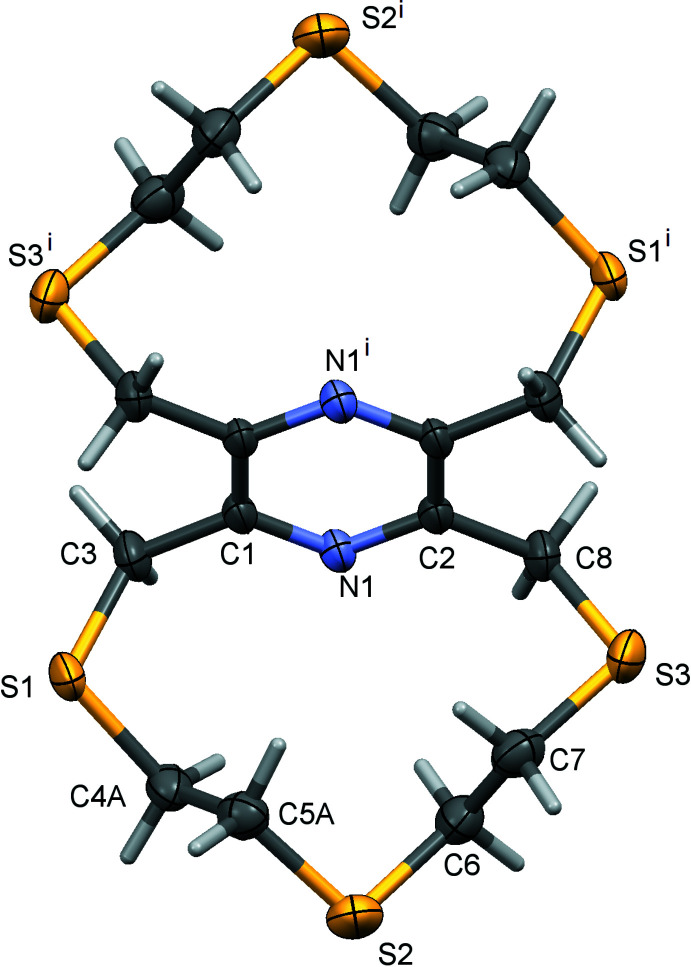
A view of the mol­ecular structure of compound **I**, the regioisomer *m*-bis L1, with atom labelling for the asymmetric unit [symmetry code: (i) −*x*, −*y*, −*z* + 1]. Displacement ellipsoids are drawn at the 50% probability level. For clarity, the minor components of the disordered atoms in the chains have been omitted.

**Figure 2:**
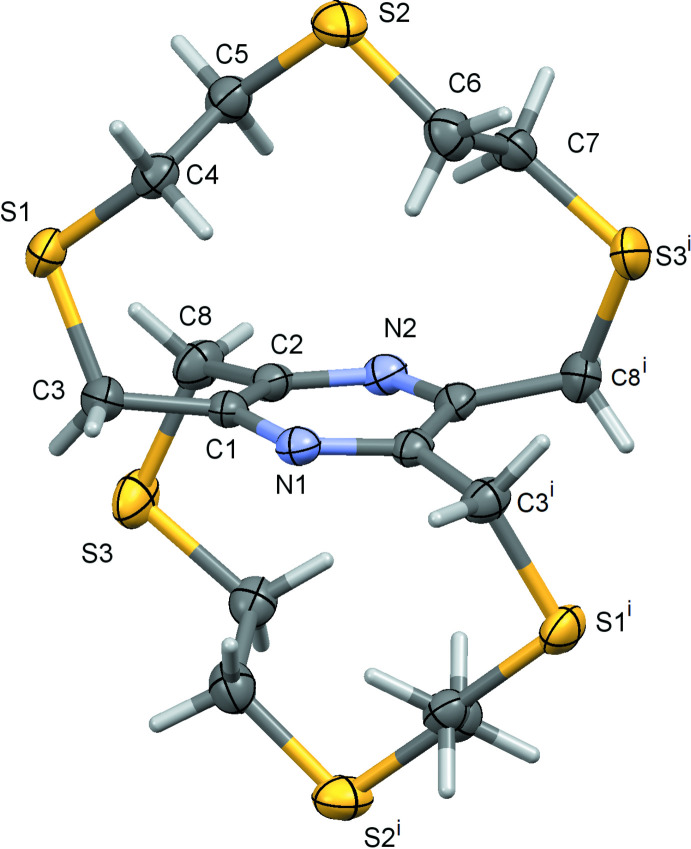
A view of the mol­ecular structure of compound **II**, the regioisomer *p*-bis L1, with atom labelling for the asymmetric unit [symmetry code: (i) −*x* + 2, *y*, −*z* + 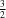]. Displacement ellipsoids are drawn at the 50% probability level.

The title thia­pyrazino­phanes, 2,5,8,11,14,17-hexa­thia-[9.9](2,6,3,5)-pyrazino­phane (**I**) and 2,5,8,11,14,17-hexa­thia-[9.9](2,5,3,6)-pyrazino­phane (**II**), are regioisomers; *m*-bis L1 and *p*-bis L1, respectively. Both compounds crystallize with half a mol­ecule in the asymmetric unit. The whole mol­ecule of **I** is generated by inversion symmetry, with the pyrazine ring being located about a center of symmetry (Fig. 1 ▸). The whole mol­ecule of **II** is generated by twofold rotation symmetry, with the pyrazine N atoms, N1 and N2, being located on the twofold rotation axis (Fig. 2 ▸). Both compounds have a central rigid tetra-2,3,5,6-methyl­ene pyrazine unit with two –S—CH_2_—CH_2_—S—CH_2_—CH_2_—S– chains linking the methyl­ene C atoms C3 and C8 [and C3^i^ and C8^i^; symmetry code: (i) −*x*, −*y*, −*z* + 1] on the pyrazine ring of **I** (Fig. 1 ▸), and linking the methyl­ene C atoms C3 and C8^i^ [C3^i^ and C8; here symmetry code: (i) −*x* + 2, *y*, −*z* + 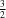] on the pyrazine ring of **II** (Fig. 2 ▸).

In **I** there are intra­molecular C—H⋯S contacts present (Table 1 ▸) but none in the mol­ecule of **II**. The pyrazine ring in **I** is planar (r.m.s. deviation = 0.003 Å), while in **II** it has a flat twist-boat conformation [puckering parameters: amplitude *Q* = 0.1158 (15) Å, θ = 90.0 (7)°, φ = 270.0 (6)°; r.m.s. deviation = 0.067 Å). In **I** atoms C4 and C5 of the –S—CH_2_—CH_2_—S—CH_2_—CH_2_—S– chain are disordered over two positions. They were refined with a fixed occupancy ratio (C4*A*:C4*B* and C5*A*:C5*B*) of 0.85:0.15.

## Supra­molecular features   

**Figure 3:**
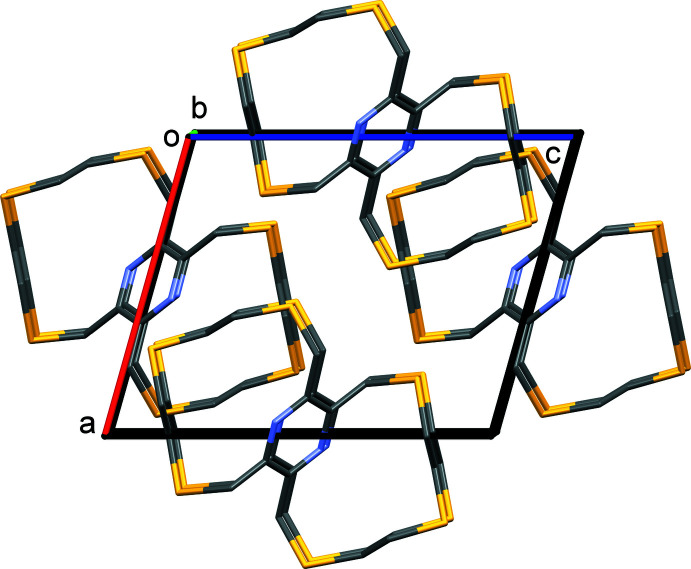
A view along the *b* axis of the crystal packing of **I**. For clarity, the minor components of the disordered atoms in the chains and the H atoms have been omitted.

**Figure 4:**
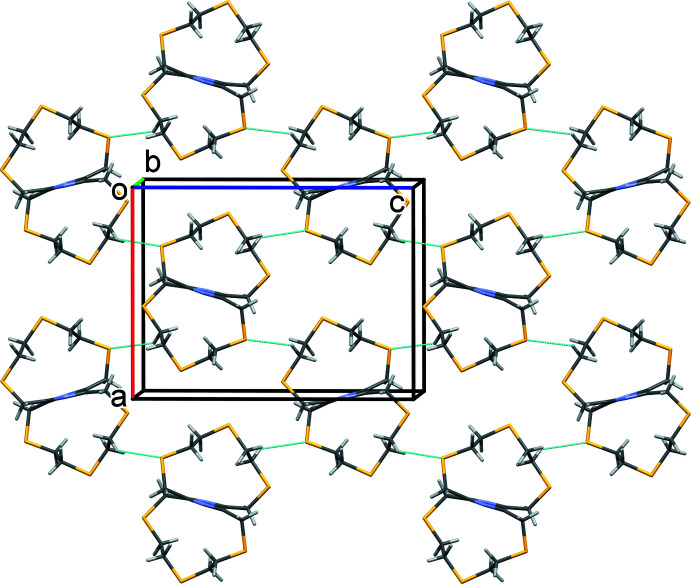
A view along the *b* axis of the crystal packing of **II**, with the C—H⋯S hydrogen bonds (Table 2 ▸) shown as dashed lines.

In the crystal of **I**, mol­ecules pack in layers that lie parallel to the (10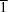) plane, as shown in Fig. 3 ▸. In the crystal of **II**, mol­ecules are linked by C—H⋯S hydrogen bonds, forming corrugated layers that lie parallel to the *ac* plane (Table 2 ▸ and Fig. 4 ▸). There are no significant inter-layer inter­actions present in the crystals of either compound.

## Hirshfeld surface analyses, two-dimensional fingerprint plots and energy frameworks for I (*m*-bis L1) and II (*p*-bis L1).   

The Hirshfeld surface analysis (Spackman & Jayatilaka, 2009 ▸), the associated two-dimensional fingerprint plots and the calculation of the energy frameworks (McKinnon *et al.*, 2007 ▸; Turner *et al.*, 2015 ▸) were performed with *CrystalExplorer17.5* (Turner *et al.*, 2017 ▸), following the protocol of Tiekink and collaborators (Tan *et al.*, 2019 ▸). The Hirshfeld surface is colour-mapped with the normalized contact distance, *d*_norm_, from red (distances shorter than the sum of the van der Waals radii) through white to blue (distances longer than the sum of the van der Waals radii). The energy frameworks are represented by cylinders joining the centroids of mol­ecular pairs using red, green and blue colour codes for the *E*_elect_ (electrostatic potential forces), *E*_disp_ (dispersion forces) and *E*_total_ (total energy) energy components, respectively. The radius of the cylinder is proportional to the magnitude of the inter­action energy.

**Figure 5:**
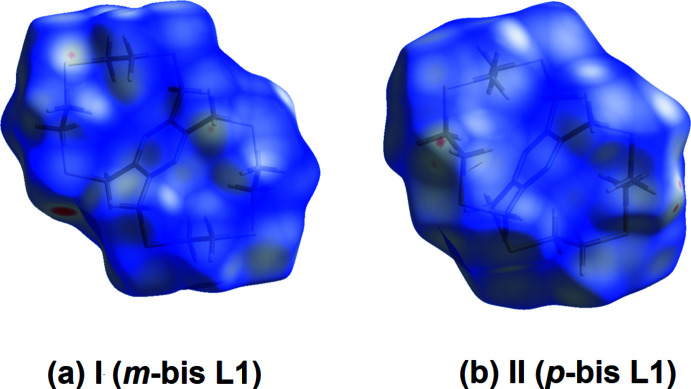
(*a*) The Hirshfeld surface of **I**, mapped over *d*_norm_ in the colour range −0.1136 to 1.0310 a.u., (*b*) the Hirshfeld surface of **II**, mapped over *d*_norm_ in the colour range −0.0862 to 1.1988 a.u.

A summary of the short inter­atomic contacts in **I** (*m*-bis L1) and **II** (*p*-bis L1) is given in Table 3 ▸. The Hirshfeld surfaces of **I** and **II** mapped over *d*_norm_, are given in Fig. 5 ▸ *a* and *b*, respectively. The faint red spots indicate that short contacts are significant in the crystal packing of both compounds.

**Figure 6:**
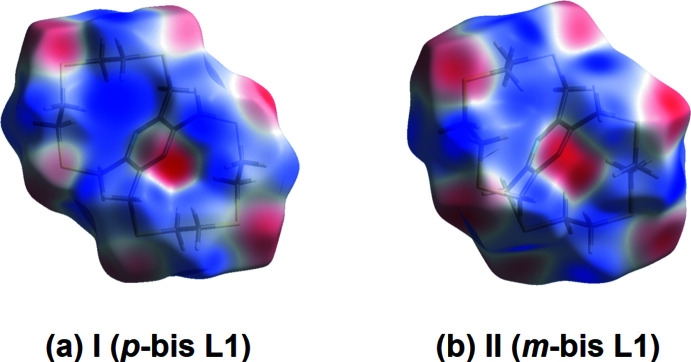
(*a*) The Hirshfeld surface of **I**, mapped over the calculated electrostatic potential in the range −0.0488 to +0.0302 atomic units, (*b*) the Hirshfeld surface of **II**, mapped over the calculated electrostatic potential in the range −0.0393 to +0.0283 atomic units. (The red and blue regions represent negative and positive electrostatic potentials, respectively.)

The Hirshfeld surfaces mapped over the calculated electrostatic potential for **I** and **II**, given in Fig. 6 ▸ *a* and *b*, respectively, are very similar. The red and blue regions represent negative and positive electrostatic potentials, respectively. The red regions around the sulfur atoms indicate their participation in the C—H⋯S contacts (see Table 3 ▸).

**Figure 7:**
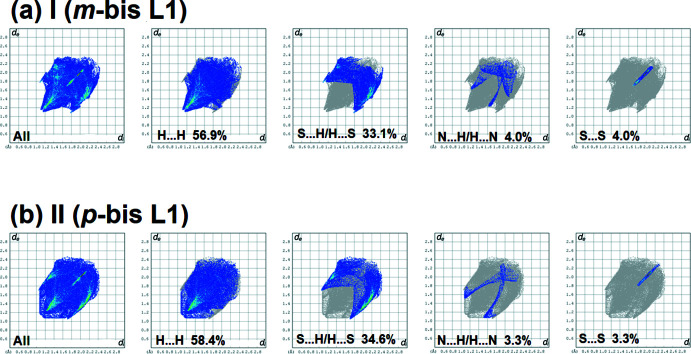
(*a*) The full two-dimensional fingerprint plot for **I**, and the fingerprint plots delineated into H⋯H, S⋯H/H⋯S, N⋯H/H⋯N and S⋯S contacts, (*b*) the full two-dimensional fingerprint plot for **II**, and the fingerprint plots delineated into H⋯H, S⋯H/H⋯S, N⋯H/H⋯N and S⋯S contacts.

The full two-dimensional fingerprint plots for **I** and **II** are given in Fig. 7 ▸. The principal inter­atomic inter­actions for **I** (Fig. 7 ▸ *a*) are delineated into H⋯H at 56.9%, S⋯H/H⋯S at 33.1%, N⋯H/H⋯N at 4.0% and S⋯S at 4.0% contacts. These values are very similar to those for **II** where the principal inter­atomic inter­actions (Fig. 7 ▸ *b*) are delineated into H⋯H at 58.4%, S⋯H/H⋯S at 34.6%, N⋯H/H⋯N at 3.3%, and S⋯S at 3.3% contacts.

**Figure 8:**
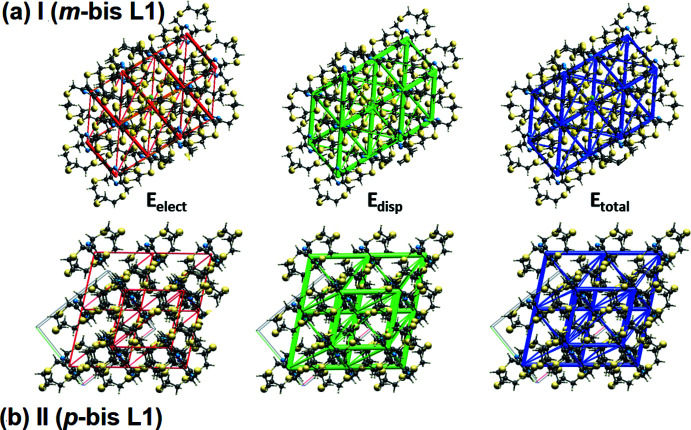
(*a*) The energy frameworks for **I** viewed down the *b*-axis direction, (*b*) the energy frameworks for **II** viewed down the *c*-axis direction: comprising, *E*_elect_ (electrostatic potential forces), *E*_disp_ (dispersion forces) and *E*_total_ (total energy) for a cluster about a reference mol­ecule.

For both **I** and **II** the inter­atomic contacts are dominated by dispersion forces, as can be seen when comparing the electrostatic potential (*E*_elect_) and dispersion (*E*_disp_) energy frameworks in Fig. 8 ▸ *a* and *b*, respectively. The energy frameworks (Fig. 8 ▸) were adjusted to the same scale factor of 80 with a cut-off value of 5 kJ mol^−1^ within a radius of 6 Å about a central mol­ecule, and were obtained using the wave function calculated at the HF/3-21G level theory.

## Database survey   

A search of the Cambridge Structural Database (Version 5.41, last update March 2020; Groom *et al.*, 2016 ▸) for benzene analogues of **L1** gave no hits for either *m*-bis or *p*-bis hexa­thia­benzeno­phanes. However, the structure of the *o*-bis hexa­thia­benzeno­phane has been reported; 2,5,8,17,20,23-hexa­thia­(9)(1,2)(9)(4,5)cyclo­phane (CSD refcode YESNEP: Loeb & Shimizu, 1994 ▸). There are also reports of the structures of two polymorphs of the *o*-mono tri­thia­benzeno­phane, 2,5,8-tri­thia­(9)-*o*-benzeno­phane (POCPAY: Klinga *et al.*, 1994 ▸; VEYNIW01: Lockhart *et al.*, 1992 ▸) and that of the *m*-mono tri­thia­benzeno­phane, 2,5,8-tri­thia­(9)-*m*-benzeno­phane (VEYNES: De Groot & Loeb, 1990 ▸). The coordination chemistry of all three compounds has been studied, especially that of YESNEP (*o-*bis hexa­thia­benzeno­phane). Binuclear complexes were obtained with copper(II) salts and AgBF_4_ (Loeb & Shimizu, 1991 ▸; 1993 ▸), with all six S atoms involved in coordination.

## Synthesis and crystallization   

**Synthesis of 2,5,8,11,14,17-hexa­thia-[9.9](2,6,3,5)-pyrazino­phane (I)**: A 500 ml three-necked flask was equipped with a reflux condenser, a 50 ml addition funnel, and a magnetic stirring bar. The entire system was purged and kept under an atmosphere of nitro­gen using vacuum line techniques. KOH (0.62 g, 11 mmol) was dissolved in a solution of MeOH/CH_2_Cl_2_ (250 ml, 1/1 *v*/*v*) in the flask. To this well-stirred mixture was added slowly and dropwise through the addition funnel, a solution of 1 g (2.21 mmol) of 2,3,5,6-tetra­kis(bromo­meth­yl)pyrazine (Ferigo *et al.*, 1994 ▸; Assoumatine & Stoeckli-Evans, 2014 ▸) and *bis*-(2-mercaptoeth­yl)sulfide (0.6 ml, 4.42 mmol, 95%) dissolved in CH_2_Cl_2_ (25 ml), at a rate of *ca* 10 ml h^−1^. The mixture was stirred for a further 20 h. The reaction mixture was taken to dryness on a rotary evaporator. The residue was extracted into CH_2_Cl_2_ (300 ml), washed with water (3 × 30 ml), dried over anhydrous MgSO_4_, filtered and then evaporated to dryness. The resultant yellowish solid was chromatographed over deactivated silica gel using CH_2_Cl_2_ as eluent. The main eluted fraction was evaporated to give a white solid, which was dried under vacuum to obtain 0.42 g (43% yield) of pure **L1** (m.p. 581–584 K, with decomposition). Slow evaporation of a CHCl_3_ solution of **L1** gave colourless rod-like crystals of **I**, the *m*-bis L1 regioisomer, after *ca* one month. ^1^H NMR (CDCl_3_, 400 MHz): δ = 4.17 (*s*, 8H, Pz-CH_2_-S), 2.73–2.49 (*m*, 16H, S–CH_2_–CH_2_–S) ppm. ^13^C NMR (CDCl_3_, 100 MHz): δ = 149.55, 32.12, 32.08, 30.85 ppm. Analysis for C_16_H_24_N_2_S_6_ (*M*_r_ = 436.78 g mol^−1^). Calculated (%): C 44.00, H 5.55, N 6.42, S 44.13. Found (%): C 43.48, H 5.25, N 6.40, S 44.34. MS (EI, 70 eV), *m*/*z*: 436 ([*M* ^+^]. IR (KBr disc, cm^−1^): ν = 2930 s, 1423 vs, 1397 vs, 1189 s, 795 ms, 760 ms, 689 ms, 482 ms.

**Synthesis of 2,5,8,11,14,17-hexa­thia-[9.9](2,5,3,6)-pyrazino­phane (II):** Pale-yellow block-like crystals of compound **II** were obtained unexpectedly during a complexation reaction of **L1** with ZnI_2_ (Assoumatine, 1999 ▸). It is difficult to imagine that the complexation reaction resulted in the transformation of *m*-bis L1 (**I**) into *p*-bis L1 (**II**). We believe it is more likely that the latter was obtained in small qu­anti­ties during the various syntheses of **L1** and was present in the main eluted fraction used subsequently for the complexation reaction. There are no analytical or spectroscopic data available for this compound.

## Refinement   

Crystal data, data collection and structure refinement details are summarized in Table 4 ▸. The C-bound H atoms were included in calculated positions and treated as riding on their parent C atom: C—H = 0.98 Å with *U*_iso_(H) = 1.2*U*_eq_(C). In **I** atoms C4 and C5 of the –CH_2_—S—CH_2_—CH_2_—S—CH_2_—CH_2_—S—CH_2_– chain are disordered over two positions. They were refined with a fixed occupancy ratio (C4*A*:C4*B* and C5*A*:C5*B*) of 0.85:0.15.

Intensity data were measured using a STOE IPDS-1 one-circle diffractometer. For the monoclinic system often only 93% of the Ewald sphere is accessible, which explains why the B alert diffrn_reflns_laue_measured_fraction_full value low at 0.957 for compound **I** is given. This involves 76 random reflections out of the expected 1765 for the IUCr cutoff limit of sin θ/λ = 0.60 for **I**.

## Supplementary Material

Crystal structure: contains datablock(s) I, II, Global. DOI: 10.1107/S2056989020007057/pk2633sup1.cif

Structure factors: contains datablock(s) I. DOI: 10.1107/S2056989020007057/pk2633Isup2.hkl

Structure factors: contains datablock(s) II. DOI: 10.1107/S2056989020007057/pk2633IIsup3.hkl

CCDC references: 2005740, 2005739

Additional supporting information:  crystallographic information; 3D view; checkCIF report

## Figures and Tables

**Table 1:** Hydrogen-bond geometry (Å, °) for **I** [Chem scheme1]

*D*—H⋯*A*	*D*—H	H⋯*A*	*D*⋯*A*	*D*—H⋯*A*
C3—H3*B*⋯S3^i^	0.98	2.77	3.524 (3)	134

Symmetry code: (i) 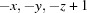.

**Table 2:** Hydrogen-bond geometry (Å, °) for **II** [Chem scheme1]

*D*—H⋯*A*	*D*—H	H⋯*A*	*D*⋯*A*	*D*—H⋯*A*
C4—H4*A*⋯S3^i^	0.98	2.83	3.581 (2)	134

Symmetry code: (i) 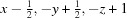.

**Table 3:** Table 3 ▸. Short inter­atomic contacts^*a*^ (Å) for **I** (*m*-bis L1) and **II** (*p*-bis L1)

Atom1⋯Atom2	Length	Length − VdW	Symm. op. 1	Symm. op. 2
**I**				
S1⋯S1	3.3938 (11)	−0.206	−*x*, −*y*, 1 − *z*	−1 + *x*, *y*, *z*
S3⋯S1	3.5135 (11)	−0.086	*x*, *y*, *z*	−1 + *x*, *y*, *z*
H3*A*⋯S2	2.969	−0.031	−*x*, −*y*, 1 − *z*	− 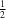 + *x*, − 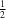 − *y*, − 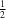 + *z*
H8*A*⋯S1	3.007	0.007	*x*, *y*, *z*	−1 + *x*, *y*, *z*
H6*A*⋯H7*A*	2.415	0.015	−*x*, −*y*, 1 − *z*	*x*, *y*, −1 + *z*
H4*A*2⋯H5*A*2	2.487	0.087	−*x*, −*y*, 1 − *z*	− 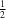 + *x*, − 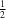 − *y*, − 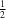 + *z*
				
**II**				
H4*A*⋯S3	2.828	−0.172	−1 + *x*, *y*, *z*	− 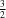 + *x*, 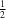 − *y*, 1 − *z*
C3⋯H7*A*	2.842	−0.058	1 − *x*, *y*, 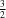 − *z*	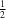 − *x*, − 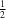 + *y*, *z*
H3*A*⋯H7*A*	2.345	−0.055	1 − *x*, *y*, 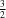 − *z*	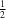 − *x*, − 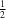 + *y*, *z*
N1⋯H7*A*	2.700	−0.050	−1 + *x*, *y*, *z*	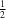 − *x*, − 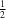 + *y*, *z*
S1⋯S3	3.6360 (6)	0.036	−1 + *x*, *y*, *z*	− 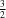 + *x*, 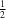 − *y*, 1 − *z*
H8*B*⋯H8*B*	2.444	0.044	−1 + *x*, *y*, *z*	1 − *x*, 1 − *y*, 1 − *z*
S3⋯H5*A*	3.072	0.072	−1 + *x*, *y*, *z*	1 − *x*, 1 − *y*, 1 − *z*
C1⋯H7*A*	2.976	0.076	1 − *x*, *y*, 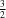 − *z*	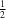 − *x*, − 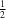 + *y*, *z*
C4⋯S3	3.5806 (16)	0.081	−1 + *x*, *y*, *z*	− 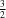 + *x*, 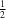 − *y*, 1 − *z*

Note: (*a*) Values were calculated using *Mercury* (Macrae *et al.*, 2020 ▸).

**Table 4:** Experimental details

	**I**	**II**
Crystal data		
Chemical formula	C_16_H_24_N_2_S_6_	C_16_H_24_N_2_S_6_
*M*_r_	436.73	436.73
Crystal system, space group	Monoclinic, *P*2_1_/*n*	Orthorhombic, *P* *b* *c* *n*
Temperature (K)	223	223
*a*, *b*, *c* (Å)	9.4078 (7), 9.2511 (7), 11.6953 (8)	12.2613 (8), 9.9564 (6), 16.2828 (12)
α, β, γ (°)	90, 105.722 (8), 90	90, 90, 90
*V* (Å^3^)	979.79 (13)	1987.8 (2)
*Z*	2	4
Radiation type	Mo *K*α	Mo *K*α
μ (mm^−1^)	0.70	0.69
Crystal size (mm)	0.40 × 0.15 × 0.15	0.25 × 0.20 × 0.10

Data collection		
Diffractometer	Stoe IPDS 1	Stoe IPDS 1
Absorption correction	Multi-scan (*MULABS*; Spek, 2020 ▸)	Multi-scan (*MULABS*; Spek, 2020 ▸)
*T*_min_, *T*_max_	0.964, 1.000	0.915, 1.000
No. of measured, independent and observed [*I* > 2σ(*I*)] reflections	7493, 1812, 1469	12271, 1927, 1521
*R*_int_	0.030	0.033
(sin θ/λ)_max_ (Å^−1^)	0.613	0.615

Refinement		
*R*[*F* ^2^ > 2σ(*F* ^2^)], *wR*(*F* ^2^), *S*	0.042, 0.108, 1.03	0.025, 0.064, 0.97
No. of reflections	1812	1927
No. of parameters	127	110
H-atom treatment	H-atom parameters constrained	H-atom parameters constrained
Δρ_max_, Δρ_min_ (e Å^−3^)	0.80, −0.35	0.27, −0.19

Computer programs: *EXPOSE*, *CELL* (Stoe & Cie, 1998 ▸) and *INTEGRATE* (Stoe & Cie, 1998 ▸), *SHELXS97* (Sheldrick, 2008 ▸), *SHELXL2018/3* (Sheldrick, 2015 ▸), *Mercury* (Macrae *et al.*, 2020 ▸), *PLATON* (Spek, 2020 ▸) and *publCIF* (Westrip, 2010 ▸).

## supplementary crystallographic information

### 2,5,8,11,14,17-Hexathia-[9.9](2,6,3,5)-pyrazinophane (I) Crystal data

**Table:**

C_16_H_24_N_2_S_6_	*F*(000) = 460
*M**_r_* = 436.73	*D*_x_ = 1.480 Mg m^−^^3^
Monoclinic, *P*2_1_/*n*	Mo *K*α radiation, λ = 0.71073 Å
*a* = 9.4078 (7) Å	Cell parameters from 5000 reflections
*b* = 9.2511 (7) Å	θ = 2.9–25.8°
*c* = 11.6953 (8) Å	µ = 0.70 mm^−^^1^
β = 105.722 (8)°	*T* = 223 K
*V* = 979.79 (13) Å^3^	Rod, colourless
*Z* = 2	0.40 × 0.15 × 0.15 mm

### 2,5,8,11,14,17-Hexathia-[9.9](2,6,3,5)-pyrazinophane (I) Data collection

**Table:**

STOE IPDS 1 diffractometer	1812 independent reflections
Radiation source: fine-focus sealed tube	1469 reflections with *I* > 2σ(*I*)
Plane graphite monochromator	*R*_int_ = 0.030
φ rotation scans	θ_max_ = 25.8°, θ_min_ = 2.9°
Absorption correction: multi-scan (MULABS; Spek, 2020)	*h* = −11→11
*T*_min_ = 0.964, *T*_max_ = 1.000	*k* = −11→11
7493 measured reflections	*l* = −14→14

### 2,5,8,11,14,17-Hexathia-[9.9](2,6,3,5)-pyrazinophane (I) Refinement

**Table:**

Refinement on *F*^2^	Primary atom site location: structure-invariant direct methods
Least-squares matrix: full	Secondary atom site location: difference Fourier map
*R*[*F*^2^ > 2σ(*F*^2^)] = 0.042	Hydrogen site location: inferred from neighbouring sites
*wR*(*F*^2^) = 0.108	H-atom parameters constrained
*S* = 1.03	*w* = 1/[σ^2^(*F*_o_^2^) + (0.0489*P*)^2^ + 1.0972*P*] where *P* = (*F*_o_^2^ + 2*F*_c_^2^)/3
1812 reflections	(Δ/σ)_max_ < 0.001
127 parameters	Δρ_max_ = 0.80 e Å^−^^3^
0 restraints	Δρ_min_ = −0.35 e Å^−^^3^

### 2,5,8,11,14,17-Hexathia-[9.9](2,6,3,5)-pyrazinophane (I) Special details

**Table:**

Geometry. All esds (except the esd in the dihedral angle between two l.s. planes) are estimated using the full covariance matrix. The cell esds are taken into account individually in the estimation of esds in distances, angles and torsion angles; correlations between esds in cell parameters are only used when they are defined by crystal symmetry. An approximate (isotropic) treatment of cell esds is used for estimating esds involving l.s. planes.

### 2,5,8,11,14,17-Hexathia-[9.9](2,6,3,5)-pyrazinophane (I) Fractional atomic coordinates and isotropic or equivalent isotropic displacement parameters (Å^2^)

**Table:**

	*x*	*y*	*z*	*U*_iso_*/*U*_eq_	Occ. (<1)
S1	0.43465 (7)	0.06030 (10)	0.61310 (7)	0.0448 (2)	
S2	0.29210 (9)	0.05009 (10)	0.94357 (7)	0.0506 (3)	
S3	−0.18897 (9)	0.04529 (10)	0.76708 (7)	0.0455 (2)	
N1	0.0478 (2)	0.1229 (2)	0.56726 (18)	0.0282 (5)	
C1	0.1307 (3)	0.0623 (3)	0.5033 (2)	0.0275 (5)	
C2	−0.0828 (3)	0.0624 (3)	0.5644 (2)	0.0273 (5)	
C3	0.2743 (3)	0.1366 (3)	0.5079 (3)	0.0364 (6)	
H3A	0.265885	0.238486	0.528088	0.044*	
H3B	0.290236	0.133282	0.428547	0.044*	
C4A	0.3996 (4)	0.1160 (4)	0.7527 (3)	0.0361 (8)	0.85
H4A1	0.490907	0.153803	0.806259	0.043*	0.85
H4A2	0.325885	0.193463	0.737391	0.043*	0.85
C5A	0.3448 (4)	−0.0092 (4)	0.8112 (3)	0.0366 (8)	0.85
H5A1	0.422534	−0.082600	0.833774	0.044*	0.85
H5A2	0.259364	−0.053204	0.754762	0.044*	0.85
C4B	0.373 (2)	−0.010 (2)	0.7494 (17)	0.032 (4)	0.15
H4B1	0.272978	−0.051440	0.724257	0.039*	0.15
H4B2	0.440973	−0.083717	0.793123	0.039*	0.15
C5B	0.375 (2)	0.122 (2)	0.823 (2)	0.038 (4)	0.15
H5B1	0.315371	0.199332	0.777647	0.045*	0.15
H5B2	0.476508	0.156417	0.857125	0.045*	0.15
C6	0.1059 (3)	0.1134 (3)	0.8751 (3)	0.0405 (7)	
H6A	0.072122	0.171931	0.932460	0.049*	
H6B	0.106432	0.175041	0.807071	0.049*	
C7	0.0005 (4)	−0.0096 (3)	0.8340 (2)	0.0413 (7)	
H7A	0.002992	−0.072264	0.902097	0.050*	
H7B	0.034589	−0.066909	0.776044	0.050*	
C8	−0.1725 (3)	0.1385 (3)	0.6345 (2)	0.0349 (6)	
H8A	−0.271975	0.154582	0.582373	0.042*	
H8B	−0.128240	0.233573	0.657957	0.042*	

### 2,5,8,11,14,17-Hexathia-[9.9](2,6,3,5)-pyrazinophane (I) Atomic displacement parameters (Å^2^)

**Table:**

	*U*^11^	*U*^22^	*U*^33^	*U*^12^	*U*^13^	*U*^23^
S1	0.0174 (3)	0.0797 (6)	0.0381 (4)	0.0007 (3)	0.0089 (3)	−0.0106 (4)
S2	0.0430 (5)	0.0790 (6)	0.0271 (4)	0.0006 (4)	0.0050 (3)	0.0023 (4)
S3	0.0354 (4)	0.0713 (6)	0.0365 (4)	−0.0095 (4)	0.0212 (3)	−0.0040 (4)
N1	0.0184 (10)	0.0387 (12)	0.0281 (10)	0.0012 (9)	0.0071 (9)	0.0016 (9)
C1	0.0159 (11)	0.0415 (14)	0.0254 (12)	0.0008 (10)	0.0059 (10)	0.0052 (10)
C2	0.0177 (11)	0.0415 (14)	0.0234 (12)	0.0025 (10)	0.0066 (9)	0.0041 (10)
C3	0.0209 (12)	0.0500 (17)	0.0405 (15)	−0.0066 (11)	0.0120 (12)	−0.0037 (13)
C4A	0.0294 (17)	0.043 (2)	0.0362 (19)	−0.0055 (14)	0.0089 (16)	−0.0103 (15)
C5A	0.036 (2)	0.037 (2)	0.035 (2)	0.0039 (14)	0.0060 (17)	0.0004 (16)
C4B	0.022 (10)	0.050 (13)	0.020 (9)	−0.005 (8)	−0.002 (8)	0.004 (8)
C5B	0.034 (11)	0.036 (11)	0.045 (12)	−0.002 (8)	0.014 (10)	−0.011 (9)
C6	0.0411 (16)	0.0496 (18)	0.0337 (14)	−0.0030 (13)	0.0153 (13)	−0.0045 (12)
C7	0.0492 (18)	0.0459 (16)	0.0306 (14)	−0.0032 (14)	0.0138 (13)	0.0029 (12)
C8	0.0222 (12)	0.0481 (16)	0.0369 (14)	0.0031 (11)	0.0126 (11)	−0.0026 (12)

### 2,5,8,11,14,17-Hexathia-[9.9](2,6,3,5)-pyrazinophane (I) Geometric parameters (Å, º)

**Table:**

S1—C3	1.811 (3)	C4A—H4A1	0.9800
S1—C4A	1.825 (4)	C4A—H4A2	0.9800
S1—C4B	1.95 (2)	C5A—H5A1	0.9800
S2—C6	1.814 (3)	C5A—H5A2	0.9800
S2—C5A	1.833 (4)	C4B—C5B	1.49 (3)
S2—C5B	1.90 (2)	C4B—H4B1	0.9800
S3—C7	1.815 (3)	C4B—H4B2	0.9800
S3—C8	1.817 (3)	C5B—H5B1	0.9800
N1—C1	1.341 (3)	C5B—H5B2	0.9800
N1—C2	1.342 (3)	C6—C7	1.500 (4)
C1—C2^i^	1.402 (4)	C6—H6A	0.9800
C1—C3	1.504 (3)	C6—H6B	0.9800
C2—C8	1.501 (4)	C7—H7A	0.9800
C3—H3A	0.9800	C7—H7B	0.9800
C3—H3B	0.9800	C8—H8A	0.9800
C4A—C5A	1.505 (5)	C8—H8B	0.9800
			
C3—S1—C4A	100.29 (15)	C5B—C4B—S1	103.8 (15)
C3—S1—C4B	107.8 (6)	C5B—C4B—H4B1	111.0
C6—S2—C5A	100.00 (15)	S1—C4B—H4B1	111.0
C6—S2—C5B	95.9 (6)	C5B—C4B—H4B2	111.0
C7—S3—C8	101.53 (13)	S1—C4B—H4B2	111.0
C1—N1—C2	118.6 (2)	H4B1—C4B—H4B2	109.0
N1—C1—C2^i^	120.8 (2)	C4B—C5B—S2	101.5 (15)
N1—C1—C3	116.2 (2)	C4B—C5B—H5B1	111.5
C2^i^—C1—C3	123.0 (2)	S2—C5B—H5B1	111.5
N1—C2—C1^i^	120.6 (2)	C4B—C5B—H5B2	111.5
N1—C2—C8	115.9 (2)	S2—C5B—H5B2	111.5
C1^i^—C2—C8	123.5 (2)	H5B1—C5B—H5B2	109.3
C1—C3—S1	114.9 (2)	C7—C6—S2	111.8 (2)
C1—C3—H3A	108.5	C7—C6—H6A	109.3
S1—C3—H3A	108.5	S2—C6—H6A	109.3
C1—C3—H3B	108.5	C7—C6—H6B	109.3
S1—C3—H3B	108.5	S2—C6—H6B	109.3
H3A—C3—H3B	107.5	H6A—C6—H6B	107.9
C5A—C4A—S1	110.9 (3)	C6—C7—S3	114.4 (2)
C5A—C4A—H4A1	109.5	C6—C7—H7A	108.7
S1—C4A—H4A1	109.5	S3—C7—H7A	108.7
C5A—C4A—H4A2	109.5	C6—C7—H7B	108.7
S1—C4A—H4A2	109.5	S3—C7—H7B	108.7
H4A1—C4A—H4A2	108.0	H7A—C7—H7B	107.6
C4A—C5A—S2	111.0 (3)	C2—C8—S3	115.73 (19)
C4A—C5A—H5A1	109.4	C2—C8—H8A	108.3
S2—C5A—H5A1	109.4	S3—C8—H8A	108.3
C4A—C5A—H5A2	109.4	C2—C8—H8B	108.3
S2—C5A—H5A2	109.4	S3—C8—H8B	108.3
H5A1—C5A—H5A2	108.0	H8A—C8—H8B	107.4
			
C2—N1—C1—C2^i^	−0.6 (4)	C6—S2—C5A—C4A	83.6 (3)
C2—N1—C1—C3	178.7 (2)	S1—C4B—C5B—S2	173.4 (8)
C1—N1—C2—C1^i^	0.6 (4)	C5A—S2—C6—C7	73.8 (2)
C1—N1—C2—C8	−177.9 (2)	C5B—S2—C6—C7	112.7 (6)
N1—C1—C3—S1	97.1 (2)	S2—C6—C7—S3	178.84 (15)
C2^i^—C1—C3—S1	−83.5 (3)	C8—S3—C7—C6	65.5 (2)
C4A—S1—C3—C1	−70.9 (2)	N1—C2—C8—S3	−109.9 (2)
C4B—S1—C3—C1	−33.9 (7)	C1^i^—C2—C8—S3	71.6 (3)
C3—S1—C4A—C5A	103.8 (3)	C7—S3—C8—C2	45.9 (2)
S1—C4A—C5A—S2	−174.34 (17)		

Symmetry code: (i) −*x*, −*y*, −*z*+1.

### 2,5,8,11,14,17-Hexathia-[9.9](2,6,3,5)-pyrazinophane (I) Hydrogen-bond geometry (Å, º)

**Table:**

*D*—H···*A*	*D*—H	H···*A*	*D*···*A*	*D*—H···*A*
C3—H3*B*···S3^i^	0.98	2.77	3.524 (3)	134

Symmetry code: (i) −*x*, −*y*, −*z*+1.

### 2,5,8,11,14,17-Hexathia-[9.9](2,5,3,6)-pyrazinophane (II) Crystal data

**Table:**

C_16_H_24_N_2_S_6_	*D*_x_ = 1.459 Mg m^−^^3^
*M**_r_* = 436.73	Mo *K*α radiation, λ = 0.71073 Å
Orthorhombic, *P**b**c**n*	Cell parameters from 5000 reflections
*a* = 12.2613 (8) Å	θ = 2.5–25.9°
*b* = 9.9564 (6) Å	µ = 0.69 mm^−^^1^
*c* = 16.2828 (12) Å	*T* = 223 K
*V* = 1987.8 (2) Å^3^	Block, pale yellow
*Z* = 4	0.25 × 0.20 × 0.10 mm
*F*(000) = 920	

### 2,5,8,11,14,17-Hexathia-[9.9](2,5,3,6)-pyrazinophane (II) Data collection

**Table:**

STOE IPDS 1 diffractometer	1927 independent reflections
Radiation source: fine-focus sealed tube	1521 reflections with *I* > 2σ(*I*)
Plane graphite monochromator	*R*_int_ = 0.033
φ rotation scans	θ_max_ = 25.9°, θ_min_ = 2.5°
Absorption correction: multi-scan (MULABS; Spek, 2020)	*h* = −15→14
*T*_min_ = 0.915, *T*_max_ = 1.000	*k* = −12→10
12271 measured reflections	*l* = −19→19

### 2,5,8,11,14,17-Hexathia-[9.9](2,5,3,6)-pyrazinophane (II) Refinement

**Table:**

Refinement on *F*^2^	Primary atom site location: structure-invariant direct methods
Least-squares matrix: full	Secondary atom site location: difference Fourier map
*R*[*F*^2^ > 2σ(*F*^2^)] = 0.025	Hydrogen site location: inferred from neighbouring sites
*wR*(*F*^2^) = 0.064	H-atom parameters constrained
*S* = 0.97	*w* = 1/[σ^2^(*F*_o_^2^) + (0.0418*P*)^2^] where *P* = (*F*_o_^2^ + 2*F*_c_^2^)/3
1927 reflections	(Δ/σ)_max_ = 0.001
110 parameters	Δρ_max_ = 0.27 e Å^−^^3^
0 restraints	Δρ_min_ = −0.19 e Å^−^^3^

### 2,5,8,11,14,17-Hexathia-[9.9](2,5,3,6)-pyrazinophane (II) Special details

**Table:**

Geometry. All esds (except the esd in the dihedral angle between two l.s. planes) are estimated using the full covariance matrix. The cell esds are taken into account individually in the estimation of esds in distances, angles and torsion angles; correlations between esds in cell parameters are only used when they are defined by crystal symmetry. An approximate (isotropic) treatment of cell esds is used for estimating esds involving l.s. planes.

### 2,5,8,11,14,17-Hexathia-[9.9](2,5,3,6)-pyrazinophane (II) Fractional atomic coordinates and isotropic or equivalent isotropic displacement parameters (Å^2^)

**Table:**

	*x*	*y*	*z*	*U*_iso_*/*U*_eq_	
S1	0.92584 (3)	0.20291 (5)	0.52666 (2)	0.03769 (13)	
S2	0.62939 (3)	0.32039 (5)	0.66508 (3)	0.04190 (13)	
S3	1.22003 (4)	0.45569 (5)	0.60156 (2)	0.04264 (14)	
N1	1.000000	0.18412 (17)	0.750000	0.0261 (4)	
N2	1.000000	0.46125 (17)	0.750000	0.0281 (4)	
C1	1.01977 (11)	0.25255 (15)	0.68044 (9)	0.0251 (3)	
C2	1.02875 (11)	0.39270 (15)	0.68267 (9)	0.0265 (3)	
C3	1.03062 (13)	0.16999 (18)	0.60363 (9)	0.0332 (4)	
H3A	1.027915	0.074691	0.618548	0.040*	
H3B	1.102268	0.187440	0.579190	0.040*	
C4	0.80303 (13)	0.19184 (16)	0.58900 (9)	0.0314 (3)	
H4A	0.750656	0.131963	0.562050	0.038*	
H4B	0.821427	0.152829	0.642492	0.038*	
C5	0.75070 (14)	0.32828 (17)	0.60188 (9)	0.0337 (4)	
H5A	0.731576	0.366522	0.548318	0.040*	
H5B	0.803729	0.388415	0.627872	0.040*	
C6	0.69013 (14)	0.31977 (17)	0.76694 (10)	0.0375 (4)	
H6A	0.636307	0.286838	0.806617	0.045*	
H6B	0.752112	0.257625	0.767392	0.045*	
C7	0.72885 (13)	0.45755 (16)	0.79340 (9)	0.0329 (4)	
H7A	0.668292	0.521440	0.789306	0.039*	
H7B	0.786870	0.487818	0.756366	0.039*	
C8	1.07313 (13)	0.47543 (17)	0.61355 (9)	0.0353 (4)	
H8A	1.056488	0.570261	0.623694	0.042*	
H8B	1.036924	0.449113	0.562359	0.042*	

### 2,5,8,11,14,17-Hexathia-[9.9](2,5,3,6)-pyrazinophane (II) Atomic displacement parameters (Å^2^)

**Table:**

	*U*^11^	*U*^22^	*U*^33^	*U*^12^	*U*^13^	*U*^23^
S1	0.0380 (2)	0.0540 (3)	0.02104 (19)	0.0029 (2)	−0.00241 (16)	−0.00421 (16)
S2	0.0272 (2)	0.0514 (3)	0.0471 (3)	0.0005 (2)	−0.00419 (18)	−0.0071 (2)
S3	0.0389 (2)	0.0636 (3)	0.0254 (2)	−0.0117 (2)	0.00714 (17)	0.00324 (19)
N1	0.0227 (8)	0.0281 (9)	0.0274 (9)	0.000	−0.0039 (7)	0.000
N2	0.0274 (9)	0.0291 (10)	0.0277 (9)	0.000	−0.0043 (7)	0.000
C1	0.0192 (7)	0.0322 (8)	0.0239 (7)	0.0024 (6)	−0.0018 (5)	−0.0003 (6)
C2	0.0235 (7)	0.0318 (8)	0.0244 (7)	0.0009 (6)	−0.0038 (6)	0.0030 (6)
C3	0.0311 (8)	0.0405 (9)	0.0280 (8)	0.0049 (7)	−0.0021 (6)	−0.0058 (7)
C4	0.0341 (8)	0.0313 (9)	0.0288 (8)	−0.0015 (7)	−0.0050 (6)	−0.0020 (6)
C5	0.0379 (8)	0.0338 (9)	0.0294 (8)	0.0011 (7)	−0.0045 (7)	0.0004 (7)
C6	0.0362 (9)	0.0393 (9)	0.0369 (8)	0.0005 (8)	0.0056 (7)	0.0040 (7)
C7	0.0322 (8)	0.0357 (9)	0.0307 (8)	0.0094 (7)	0.0019 (7)	0.0002 (7)
C8	0.0387 (9)	0.0398 (9)	0.0273 (8)	−0.0036 (7)	−0.0011 (7)	0.0076 (7)

### 2,5,8,11,14,17-Hexathia-[9.9](2,5,3,6)-pyrazinophane (II) Geometric parameters (Å, º)

**Table:**

S1—C4	1.8193 (17)	C3—H3B	0.9800
S1—C3	1.8245 (16)	C4—C5	1.517 (2)
S2—C5	1.8104 (17)	C4—H4A	0.9800
S2—C6	1.8182 (17)	C4—H4B	0.9800
S3—C7^i^	1.8218 (16)	C5—H5A	0.9800
S3—C8	1.8224 (17)	C5—H5B	0.9800
N1—C1	1.3438 (17)	C6—C7	1.514 (2)
N1—C1^i^	1.3438 (17)	C6—H6A	0.9800
N2—C2	1.3387 (17)	C6—H6B	0.9800
N2—C2^i^	1.3388 (17)	C7—H7A	0.9800
C1—C2	1.400 (2)	C7—H7B	0.9800
C1—C3	1.503 (2)	C8—H8A	0.9800
C2—C8	1.497 (2)	C8—H8B	0.9800
C3—H3A	0.9800		
			
C4—S1—C3	100.87 (7)	C4—C5—H5A	109.0
C5—S2—C6	100.49 (7)	S2—C5—H5A	109.0
C7^i^—S3—C8	103.79 (7)	C4—C5—H5B	109.0
C1—N1—C1^i^	119.07 (18)	S2—C5—H5B	109.0
C2—N2—C2^i^	118.69 (18)	H5A—C5—H5B	107.8
N1—C1—C2	119.85 (14)	C7—C6—S2	112.64 (11)
N1—C1—C3	116.11 (14)	C7—C6—H6A	109.1
C2—C1—C3	124.04 (14)	S2—C6—H6A	109.1
N2—C2—C1	120.57 (14)	C7—C6—H6B	109.1
N2—C2—C8	115.52 (14)	S2—C6—H6B	109.1
C1—C2—C8	123.88 (14)	H6A—C6—H6B	107.8
C1—C3—S1	114.28 (11)	C6—C7—S3^i^	111.45 (11)
C1—C3—H3A	108.7	C6—C7—H7A	109.3
S1—C3—H3A	108.7	S3^i^—C7—H7A	109.3
C1—C3—H3B	108.7	C6—C7—H7B	109.3
S1—C3—H3B	108.7	S3^i^—C7—H7B	109.3
H3A—C3—H3B	107.6	H7A—C7—H7B	108.0
C5—C4—S1	111.90 (11)	C2—C8—S3	112.36 (11)
C5—C4—H4A	109.2	C2—C8—H8A	109.1
S1—C4—H4A	109.2	S3—C8—H8A	109.1
C5—C4—H4B	109.2	C2—C8—H8B	109.1
S1—C4—H4B	109.2	S3—C8—H8B	109.1
H4A—C4—H4B	107.9	H8A—C8—H8B	107.9
C4—C5—S2	112.78 (12)		
			
C1^i^—N1—C1—C2	5.44 (9)	C4—S1—C3—C1	−49.44 (14)
C1^i^—N1—C1—C3	−174.39 (14)	C3—S1—C4—C5	108.79 (12)
C2^i^—N2—C2—C1	5.52 (9)	S1—C4—C5—S2	−179.11 (8)
C2^i^—N2—C2—C8	−172.62 (14)	C6—S2—C5—C4	82.34 (13)
N1—C1—C2—N2	−11.29 (19)	C5—S2—C6—C7	76.92 (13)
C3—C1—C2—N2	168.54 (12)	S2—C6—C7—S3^i^	175.79 (8)
N1—C1—C2—C8	166.69 (12)	N2—C2—C8—S3	106.65 (12)
C3—C1—C2—C8	−13.5 (2)	C1—C2—C8—S3	−71.42 (17)
N1—C1—C3—S1	116.25 (12)	C7^i^—S3—C8—C2	−42.15 (14)
C2—C1—C3—S1	−63.58 (18)		

Symmetry code: (i) −*x*+2, *y*, −*z*+3/2.

### 2,5,8,11,14,17-Hexathia-[9.9](2,5,3,6)-pyrazinophane (II) Hydrogen-bond geometry (Å, º)

**Table:**

*D*—H···*A*	*D*—H	H···*A*	*D*···*A*	*D*—H···*A*
C4—H4*A*···S3^ii^	0.98	2.83	3.581 (2)	134

Symmetry code: (ii) *x*−1/2, −*y*+1/2, −*z*+1.

## References

[bb1] Assoumatine, T. (1999). PhD Thesis, University of Neuchâtel, Switzerland.

[bb2] Assoumatine, T. & Stoeckli-Evans, H. (2014). *Acta Cryst.* E**70**, 51–53. 10.1107/S1600536814011337 PMC4158553 25249852

[bb3] Assoumatine, T. & Stoeckli-Evans, H. (2020*a*). *Acta Cryst.* E**76**, 539–546. 10.1107/S205698902000362X PMC7133031 32280500

[bb4] Assoumatine, T. & Stoeckli-Evans, H. (2020*b*). *IUCrData*, **5**, x200467. 10.1107/S2414314620004678 PMC9462211 36338305

[bb5] Assoumatine, T. & Stoeckli-Evans, H. (2020*c*). *IUCrData*, **5**, x200401. 10.1107/S2414314620004010 PMC9462195 36339478

[bb6] Assoumatine, T. & Stoeckli-Evans, H. (2020*d*). Private communications (deposition numbers 1988248 and 1988249). CCDC, Cambridge, England.

[bb7] Assoumatine, T. & Stoeckli-Evans, H. (2020*e*). *Acta Cryst.* E**76**, 984–989. 10.1107/S2056989020007161 PMC7336783 32695438

[bb8] De Groot, B. & Loeb, S. J. (1990). *Inorg. Chem.* **29**, 4084–4090.

[bb9] Ferigo, M., Bonhôte, P., Marty, W. & Stoeckli-Evans, H. (1994). *J. Chem. Soc. Dalton Trans.* pp. 1549–1554.

[bb10] Groom, C. R., Bruno, I. J., Lightfoot, M. P. & Ward, S. C. (2016). *Acta Cryst.* B**72**, 171–179. 10.1107/S2052520616003954 PMC4822653 27048719

[bb11] Kim, S., Siewe, A. D., Lee, E., Ju, H., Park, I.-H., Jung, J. H., Habata, Y. & Lee, S. S. (2018). *Cryst. Growth Des.* **18**, 2424–2431.

[bb12] Klinga, M., Kivekäs, R., Almajano, M. P., Escriche, L. & Casabó, J. F. (1994). *Z. Kristallogr. Cryst. Mater.* **209**, 560–561.

[bb13] Lockhart, J. C., Mousley, D. P., Hill, M. N. S., Tomkinson, N. P., Teixidor, F., Almajano, M. P., Escriche, L., Casabó, J. F., Sillanpää, R. & Kivekäs, R. (1992). *J. Chem. Soc. Dalton Trans.* pp. 2889–2897.

[bb14] Loeb, S. J. & Shimizu, G. K. H. (1991). *J. Chem. Soc. Chem. Commun.* pp. 1119–1121.

[bb15] Loeb, S. J. & Shimizu, G. K. H. (1993). *Inorg. Chem.* **32**, 1001–1006.

[bb16] Loeb, S. J. & Shimizu, G. K. H. (1994). *Can. J. Chem.* **72**, 1728–1734.

[bb17] Macrae, C. F., Sovago, I., Cottrell, S. J., Galek, P. T. A., McCabe, P., Pidcock, E., Platings, M., Shields, G. P., Stevens, J. S., Towler, M. & Wood, P. A. (2020). *J. Appl. Cryst.* **53**, 226–235. 10.1107/S1600576719014092 PMC6998782 32047413

[bb18] McKinnon, J. J., Jayatilaka, D. & Spackman, M. A. (2007). *Chem. Commun.* pp. 3814–3816. 10.1039/b704980c 18217656

[bb19] Sheldrick, G. M. (2008). *Acta Cryst.* A**64**, 112–122. 10.1107/S0108767307043930 18156677

[bb20] Sheldrick, G. M. (2015). *Acta Cryst.* C**71**, 3–8.

[bb21] Siewe, A. D., Kim, J.-Y., Kim, S., Park, I.-H. & Lee, S. S. (2014). *Inorg. Chem.* **53**, 393–398. 10.1021/ic402346z 24328242

[bb22] Spackman, M. A. & Jayatilaka, D. (2009). *CrystEngComm*, **11**, 19–32.

[bb23] Spek, A. L. (2020). *Acta Cryst.* E**76**, 1–11. 10.1107/S2056989019016244 PMC6944088 31921444

[bb24] Stoe & Cie (1998). *IPDS-I* Bedienungshandbuch. Stoe & Cie GmbH, Darmstadt, Germany.

[bb25] Tan, S. L., Jotani, M. M. & Tiekink, E. R. T. (2019). *Acta Cryst.* E**75**, 308–318. 10.1107/S2056989019001129 PMC6399703 30867939

[bb26] Turner, M. J., McKinnon, J. J., Wolff, S. K., Grimwood, D. J., Spackman, P. R., Jayatilaka, D. & Spackman, M. A. (2017). *CrystalExplorer17. University of Western Australia.* http://hirshfeldsurface.net

[bb27] Turner, M. J., Thomas, S. P., Shi, M. W., Jayatilaka, D. & Spackman, M. A. (2015). *Chem. Commun.* **51**, 3735–3738. 10.1039/c4cc09074h 25525647

[bb28] Westrip, S. P. (2010). *J. Appl. Cryst.* **43**, 920–925.

